# 1-[(Cyclo­propyl­meth­oxy)meth­yl]-5-ethyl-6-(4-methyl­benzyl)-1,2,3,4-tetra­hydro­pyrimidine-2,4-dione

**DOI:** 10.1107/S1600536811055681

**Published:** 2012-01-11

**Authors:** Nasser R. El-Brollosy, Ali A. El-Emam, Omar A. Al-Deeb, Seik Weng Ng

**Affiliations:** a Department of Pharmaceutical Chemistry, College of Pharmacy, King Saud University, Riyadh 11451, Saudi Arabia; b Department of Chemistry, University of Malaya, 50603 Kuala Lumpur, Malaysia; c Chemistry Department, Faculty of Science, King Abdulaziz University, PO Box 80203 Jeddah, Saudi Arabia

## Abstract

The pyrimidine ring in the title compound, C_19_H_24_N_2_O_3_, is nearly planar (r.m.s. deviation = 0.008 Å); the C atom at the 5-position deviates by 0.054 (3) Å from the mean plane and the C atom at the 6-position by 0.006 (3) Å in the opposite direction. The benzene ring is approximately perpendicular to the pyrimidine ring [dihedral angle = 83.90 (10)°]. The amino group is hydrogen-bond donor to the exocyclic O atom at the 2-position of an adjacent mol­ecule, the hydrogen bond generating an inversion dimer. The cyclo­propyl ring is disordered over two sets of sites with the major component having 71.5 (4)% occupancy.

## Related literature

For the synthesis, see: El-Brollosy *et al.* (2009 ▶).
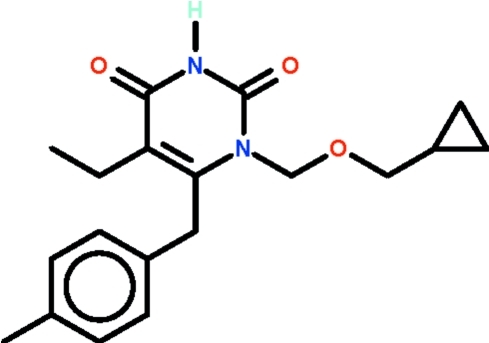


## Experimental

### 

#### Crystal data

C_19_H_24_N_2_O_3_
*M* *_r_* = 328.40
Monoclinic, 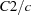

*a* = 15.6080 (13) Å
*b* = 8.1330 (6) Å
*c* = 27.496 (2) Å
β = 93.397 (7)°
*V* = 3484.2 (5) Å^3^
*Z* = 8
Mo *K*α radiation
μ = 0.09 mm^−1^
*T* = 100 K
0.25 × 0.20 × 0.15 mm

#### Data collection

Agilent SuperNova Dual diffractometer with an Atlas detector
Absorption correction: multi-scan (*CrysAlis PRO*; Agilent, 2010 ▶) *T*_min_ = 0.979, *T*_max_ = 0.987
7060 measured reflections
4006 independent reflections
2446 reflections with *I* > 2σ(*I*)
*R*_int_ = 0.044

#### Refinement

*R*[*F* ^2^ > 2σ(*F* ^2^)] = 0.061
*wR*(*F* ^2^) = 0.155
*S* = 1.06
4006 reflections
232 parameters
20 restraints
H atoms treated by a mixture of independent and constrained refinement
Δρ_max_ = 0.22 e Å^−3^
Δρ_min_ = −0.26 e Å^−3^

### 

Data collection: *CrysAlis PRO* (Agilent, 2010 ▶); cell refinement: *CrysAlis PRO*; data reduction: *CrysAlis PRO*; program(s) used to solve structure: *SHELXS97* (Sheldrick, 2008 ▶); program(s) used to refine structure: *SHELXL97* (Sheldrick, 2008 ▶); molecular graphics: *X-SEED* (Barbour, 2001 ▶); software used to prepare material for publication: *publCIF* (Westrip, 2010 ▶).

## Supplementary Material

Crystal structure: contains datablock(s) global, I. DOI: 10.1107/S1600536811055681/xu5423sup1.cif

Structure factors: contains datablock(s) I. DOI: 10.1107/S1600536811055681/xu5423Isup2.hkl

Supplementary material file. DOI: 10.1107/S1600536811055681/xu5423Isup3.cml

Additional supplementary materials:  crystallographic information; 3D view; checkCIF report

## Figures and Tables

**Table 1:** Hydrogen-bond geometry (Å, °)

*D*—H⋯*A*	*D*—H	H⋯*A*	*D*⋯*A*	*D*—H⋯*A*
N1—H1⋯O1^i^	0.89 (1)	1.93 (1)	2.816 (2)	178 (2)

Symmetry code: (i) 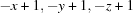.

**Fig. 1.**
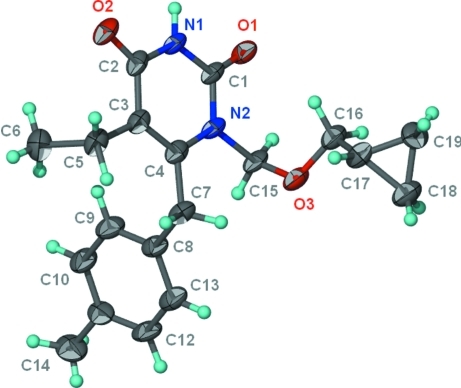
Thermal ellipsoid plot (Barbour, 2001) of C19H24N2O3 at the 70% probability level; hydrogen atoms are drawn as spheres of arbitrary radius. The disorder is not shown.

**Fig. 2.**
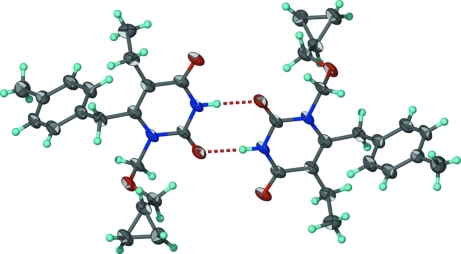
Dimeric hydrogen-bonded structure.

## supplementary crystallographic information

### Comment

The compound (Scheme I) was synthesized for an evaluation of its anti-viral activity against HIV-1 (El-Brollosy *et al.*, 2009). The pyrimidine ring is nearly planar; the C atom at the 5-position deviates by 0.054 (3) Å from the mean plane and the C atom at the 6-position by 0.006 (3) Å in the opposite direction (Fig. 1). The amino group is hydrogen-bond donor to the exocyclic O atom at the 2-position, the hydrogen bond generating a centrosymmetric dimer (Table 1, Fig. 2).

### Experimental

The compound (Scheme I) was synthesized by using a reported method (El-Brollosy *et al.*, 2009), and was recrystallized from ethanol.

### Refinement

Carbon-bound H-atoms were placed in calculated positions [C–H 0.95 to 0.98 Å, *U*_iso_(H) 1.2 to 1.5*U*_eq_(C)] and were included in the refinement in the riding model approximation.

The amino H-atom was located in a difference Fourier map, and was refined with a distance restraint of N–H 0.88±0.01 Å; its temperature factor was refined.

The cyclopropyl ring is disordered over two positions. The pair of C_methylene_–C_cyclopropyl_ bond distances were restrained to within 0.01±0.01 Å of each other, as were the bond distances of the ring itself. The temperature factors of the primed were set to those of the umprimed ones, and the anisotropic temperature factors were restrained to be nearly isotropic.

### Crystal data

**Table:**

C_19_H_24_N_2_O_3_	*F*(000) = 1408
*M**_r_* = 328.40	*D*_x_ = 1.252 Mg m^−^^3^
Monoclinic, *C*2/*c*	Mo *K*α radiation, λ = 0.71073 Å
Hall symbol: -C 2yc	Cell parameters from 1703 reflections
*a* = 15.6080 (13) Å	θ = 2.6–27.5°
*b* = 8.1330 (6) Å	µ = 0.09 mm^−^^1^
*c* = 27.496 (2) Å	*T* = 100 K
β = 93.397 (7)°	Irregular, colorless
*V* = 3484.2 (5) Å^3^	0.25 × 0.20 × 0.15 mm
*Z* = 8	

### Data collection

**Table:**

Agilent SuperNova Dual diffractometer with an Atlas detector	4006 independent reflections
Radiation source: SuperNova (Mo) X-ray Source	2446 reflections with *I* > 2σ(*I*)
Mirror	*R*_int_ = 0.044
Detector resolution: 10.4041 pixels mm^-1^	θ_max_ = 27.6°, θ_min_ = 2.6°
ω scan	*h* = −20→19
Absorption correction: multi-scan (CrysAlis PRO; Agilent, 2010)	*k* = −7→10
*T*_min_ = 0.979, *T*_max_ = 0.987	*l* = −35→21
7060 measured reflections	

### Refinement

**Table:**

Refinement on *F*^2^	Primary atom site location: structure-invariant direct methods
Least-squares matrix: full	Secondary atom site location: difference Fourier map
*R*[*F*^2^ > 2σ(*F*^2^)] = 0.061	Hydrogen site location: inferred from neighbouring sites
*wR*(*F*^2^) = 0.155	H atoms treated by a mixture of independent and constrained refinement
*S* = 1.06	*w* = 1/[σ^2^(*F*_o_^2^) + (0.0471*P*)^2^ + 1.5059*P*] where *P* = (*F*_o_^2^ + 2*F*_c_^2^)/3
4006 reflections	(Δ/σ)_max_ = 0.001
232 parameters	Δρ_max_ = 0.22 e Å^−^^3^
20 restraints	Δρ_min_ = −0.26 e Å^−^^3^

### Fractional atomic coordinates and isotropic or equivalent isotropic displacement parameters (Å^2^)

**Table:**

	*x*	*y*	*z*	*U*_iso_*/*U*_eq_	Occ. (<1)
O1	0.50675 (10)	0.53293 (18)	0.43772 (5)	0.0306 (4)	
O2	0.72573 (10)	0.51944 (19)	0.55353 (6)	0.0337 (4)	
O3	0.61688 (10)	0.75562 (18)	0.34641 (5)	0.0322 (4)	
N1	0.61833 (12)	0.5257 (2)	0.49464 (7)	0.0252 (4)	
H1	0.5798 (12)	0.508 (3)	0.5165 (7)	0.036 (7)*	
N2	0.64326 (12)	0.5688 (2)	0.41308 (6)	0.0251 (4)	
C1	0.58460 (15)	0.5419 (3)	0.44798 (8)	0.0256 (5)	
C2	0.70469 (15)	0.5330 (2)	0.51006 (8)	0.0260 (5)	
C3	0.76385 (15)	0.5573 (3)	0.47165 (8)	0.0261 (5)	
C4	0.73229 (15)	0.5764 (2)	0.42504 (8)	0.0253 (5)	
C5	0.85735 (15)	0.5580 (3)	0.48899 (9)	0.0316 (6)	
H5A	0.8648	0.6277	0.5184	0.038*	
H5B	0.8913	0.6074	0.4634	0.038*	
C6	0.89235 (16)	0.3854 (3)	0.50097 (10)	0.0394 (6)	
H6A	0.9522	0.3936	0.5136	0.059*	
H6B	0.8891	0.3180	0.4714	0.059*	
H6C	0.8580	0.3344	0.5256	0.059*	
C7	0.78826 (15)	0.6035 (3)	0.38279 (8)	0.0306 (6)	
H7A	0.8437	0.6504	0.3954	0.037*	
H7B	0.7603	0.6858	0.3606	0.037*	
C8	0.80601 (14)	0.4493 (3)	0.35365 (8)	0.0286 (5)	
C9	0.80732 (16)	0.2935 (3)	0.37445 (9)	0.0354 (6)	
H9	0.7922	0.2810	0.4072	0.043*	
C10	0.83032 (16)	0.1554 (3)	0.34834 (8)	0.0354 (6)	
H10	0.8311	0.0506	0.3636	0.042*	
C11	0.85223 (15)	0.1686 (3)	0.30026 (8)	0.0331 (6)	
C12	0.84815 (15)	0.3240 (3)	0.27877 (9)	0.0369 (6)	
H12	0.8607	0.3357	0.2456	0.044*	
C13	0.82606 (15)	0.4618 (3)	0.30501 (8)	0.0340 (6)	
H13	0.8246	0.5664	0.2896	0.041*	
C14	0.87809 (17)	0.0183 (3)	0.27244 (9)	0.0457 (7)	
H14A	0.9128	0.0518	0.2456	0.069*	
H14B	0.8265	−0.0390	0.2593	0.069*	
H14C	0.9117	−0.0554	0.2944	0.069*	
C15	0.60855 (15)	0.5928 (3)	0.36212 (8)	0.0285 (5)	
H15A	0.6393	0.5192	0.3404	0.034*	
H15B	0.5471	0.5617	0.3597	0.034*	
C16	0.55665 (16)	0.8680 (3)	0.36619 (9)	0.0326 (6)	
H16A	0.5479	0.8404	0.4006	0.039*	0.715 (4)
H16B	0.5006	0.8618	0.3474	0.039*	0.715 (4)
H16C	0.5778	0.8995	0.3995	0.039*	0.285 (4)
H16D	0.5009	0.8113	0.3686	0.039*	0.285 (4)
C17	0.5939 (2)	1.0366 (4)	0.36259 (12)	0.0311 (8)	0.715 (4)
H17	0.6480	1.0583	0.3829	0.037*	0.715 (4)
C18	0.5821 (3)	1.1319 (4)	0.31577 (14)	0.0401 (9)	0.715 (4)
H18A	0.6284	1.2083	0.3074	0.048*	0.715 (4)
H18B	0.5518	1.0771	0.2876	0.048*	0.715 (4)
C19	0.5321 (8)	1.1730 (14)	0.3578 (5)	0.0357 (18)	0.715 (4)
H19A	0.4703	1.1451	0.3558	0.043*	0.715 (4)
H19B	0.5467	1.2760	0.3756	0.043*	0.715 (4)
C17'	0.5430 (5)	1.0209 (9)	0.3360 (3)	0.0311 (8)	0.285
H17'	0.5189	1.0059	0.3018	0.037*	0.285 (4)
C18'	0.6114 (7)	1.1436 (12)	0.3439 (4)	0.0401 (9)	0.285
H18C	0.6306	1.2021	0.3149	0.048*	0.285 (4)
H18D	0.6579	1.1186	0.3688	0.048*	0.285 (4)
C19'	0.521 (2)	1.192 (4)	0.3624 (15)	0.0357 (18)	0.285
H19C	0.4865	1.2742	0.3436	0.043*	0.285 (4)
H19D	0.5140	1.1902	0.3979	0.043*	0.285 (4)

### Atomic displacement parameters (Å^2^)

**Table:**

	*U*^11^	*U*^22^	*U*^33^	*U*^12^	*U*^13^	*U*^23^
O1	0.0354 (10)	0.0381 (9)	0.0179 (8)	−0.0015 (7)	−0.0004 (7)	0.0037 (7)
O2	0.0468 (10)	0.0358 (9)	0.0176 (9)	−0.0012 (8)	−0.0050 (7)	0.0035 (7)
O3	0.0479 (10)	0.0299 (8)	0.0192 (9)	0.0014 (8)	0.0055 (7)	0.0056 (7)
N1	0.0359 (11)	0.0256 (10)	0.0140 (10)	−0.0014 (9)	0.0009 (8)	0.0030 (8)
N2	0.0356 (11)	0.0265 (9)	0.0131 (9)	−0.0029 (8)	0.0007 (8)	0.0014 (7)
C1	0.0378 (14)	0.0231 (11)	0.0158 (11)	−0.0015 (10)	0.0010 (10)	0.0027 (9)
C2	0.0398 (14)	0.0184 (10)	0.0191 (12)	0.0003 (10)	−0.0039 (10)	−0.0001 (9)
C3	0.0359 (13)	0.0212 (11)	0.0210 (12)	−0.0011 (10)	−0.0005 (10)	0.0005 (9)
C4	0.0352 (13)	0.0181 (10)	0.0226 (12)	−0.0026 (10)	0.0009 (10)	0.0005 (9)
C5	0.0388 (14)	0.0276 (12)	0.0278 (13)	−0.0026 (11)	−0.0029 (10)	0.0041 (10)
C6	0.0416 (15)	0.0349 (14)	0.0408 (16)	0.0035 (12)	−0.0052 (12)	0.0027 (11)
C7	0.0381 (14)	0.0317 (12)	0.0221 (13)	−0.0057 (11)	0.0034 (10)	0.0044 (10)
C8	0.0297 (12)	0.0378 (13)	0.0183 (12)	−0.0035 (10)	0.0008 (9)	−0.0004 (10)
C9	0.0492 (16)	0.0383 (14)	0.0193 (13)	0.0005 (12)	0.0066 (11)	−0.0012 (10)
C10	0.0508 (15)	0.0362 (13)	0.0191 (13)	0.0040 (12)	0.0018 (11)	−0.0009 (10)
C11	0.0303 (13)	0.0510 (15)	0.0177 (12)	0.0049 (11)	−0.0034 (9)	−0.0066 (11)
C12	0.0370 (14)	0.0591 (17)	0.0148 (12)	−0.0020 (12)	0.0027 (10)	0.0015 (11)
C13	0.0366 (13)	0.0446 (14)	0.0205 (13)	−0.0051 (12)	−0.0001 (10)	0.0044 (11)
C14	0.0458 (16)	0.0653 (18)	0.0256 (14)	0.0110 (14)	−0.0011 (12)	−0.0125 (13)
C15	0.0421 (14)	0.0308 (12)	0.0124 (11)	−0.0009 (11)	−0.0007 (10)	0.0016 (9)
C16	0.0436 (15)	0.0329 (13)	0.0211 (13)	0.0016 (11)	0.0018 (10)	0.0042 (10)
C17	0.0378 (18)	0.0338 (17)	0.0212 (17)	−0.0049 (15)	−0.0034 (12)	0.0026 (14)
C18	0.058 (2)	0.0379 (18)	0.024 (2)	0.0008 (18)	0.0071 (16)	0.0057 (18)
C19	0.045 (4)	0.025 (3)	0.038 (3)	0.006 (2)	0.009 (2)	−0.004 (2)
C17'	0.0378 (18)	0.0338 (17)	0.0212 (17)	−0.0049 (15)	−0.0034 (12)	0.0026 (14)
C18'	0.058 (2)	0.0379 (18)	0.024 (2)	0.0008 (18)	0.0071 (16)	0.0057 (18)
C19'	0.045 (4)	0.025 (3)	0.038 (3)	0.006 (2)	0.009 (2)	−0.004 (2)

### Geometric parameters (Å, °)

**Table:**

O1—C1	1.233 (3)	C12—C13	1.387 (3)
O2—C2	1.226 (2)	C12—H12	0.9500
O3—C15	1.401 (3)	C13—H13	0.9500
O3—C16	1.440 (3)	C14—H14A	0.9800
N1—C1	1.364 (3)	C14—H14B	0.9800
N1—C2	1.390 (3)	C14—H14C	0.9800
N1—H1	0.887 (10)	C15—H15A	0.9900
N2—C1	1.382 (3)	C15—H15B	0.9900
N2—C4	1.410 (3)	C16—C17	1.495 (4)
N2—C15	1.485 (3)	C16—C17'	1.503 (7)
C2—C3	1.457 (3)	C16—H16A	0.9900
C3—C4	1.354 (3)	C16—H16B	0.9900
C3—C5	1.508 (3)	C16—H16C	0.9900
C4—C7	1.511 (3)	C16—H16D	0.9900
C5—C6	1.535 (3)	C17—C19	1.471 (12)
C5—H5A	0.9900	C17—C18	1.505 (5)
C5—H5B	0.9900	C17—H17	1.0000
C6—H6A	0.9800	C18—C19	1.472 (12)
C6—H6B	0.9800	C18—H18A	0.9900
C6—H6C	0.9800	C18—H18B	0.9900
C7—C8	1.523 (3)	C19—H19A	0.9900
C7—H7A	0.9900	C19—H19B	0.9900
C7—H7B	0.9900	C17'—C18'	1.468 (14)
C8—C9	1.390 (3)	C17'—C19'	1.61 (3)
C8—C13	1.395 (3)	C17'—H17'	1.0000
C9—C10	1.391 (3)	C18'—C19'	1.57 (3)
C9—H9	0.9500	C18'—H18C	0.9900
C10—C11	1.389 (3)	C18'—H18D	0.9900
C10—H10	0.9500	C19'—H19C	0.9900
C11—C12	1.395 (3)	C19'—H19D	0.9900
C11—C14	1.509 (3)		
			
C15—O3—C16	114.11 (17)	H14A—C14—H14C	109.5
C1—N1—C2	126.6 (2)	H14B—C14—H14C	109.5
C1—N1—H1	114.4 (16)	O3—C15—N2	112.32 (17)
C2—N1—H1	119.0 (16)	O3—C15—H15A	109.1
C1—N2—C4	121.93 (18)	N2—C15—H15A	109.1
C1—N2—C15	117.14 (18)	O3—C15—H15B	109.1
C4—N2—C15	120.92 (18)	N2—C15—H15B	109.1
O1—C1—N1	122.0 (2)	H15A—C15—H15B	107.9
O1—C1—N2	122.3 (2)	O3—C16—C17	107.0 (2)
N1—C1—N2	115.7 (2)	O3—C16—C17'	113.0 (4)
O2—C2—N1	119.5 (2)	O3—C16—H16A	110.3
O2—C2—C3	125.1 (2)	C17—C16—H16A	110.3
N1—C2—C3	115.44 (19)	C17'—C16—H16A	133.8
C4—C3—C2	119.4 (2)	O3—C16—H16B	110.3
C4—C3—C5	126.1 (2)	C17—C16—H16B	110.3
C2—C3—C5	114.5 (2)	H16A—C16—H16B	108.6
C3—C4—N2	120.9 (2)	O3—C16—H16C	109.0
C3—C4—C7	123.4 (2)	C17—C16—H16C	73.4
N2—C4—C7	115.69 (19)	C17'—C16—H16C	109.0
C3—C5—C6	112.95 (19)	O3—C16—H16D	109.0
C3—C5—H5A	109.0	C17'—C16—H16D	109.0
C6—C5—H5A	109.0	H16C—C16—H16D	107.8
C3—C5—H5B	109.0	C19—C17—C16	116.2 (5)
C6—C5—H5B	109.0	C19—C17—C18	59.3 (6)
H5A—C5—H5B	107.8	C16—C17—C18	120.0 (3)
C5—C6—H6A	109.5	C19—C17—H17	116.3
C5—C6—H6B	109.5	C16—C17—H17	116.3
H6A—C6—H6B	109.5	C18—C17—H17	116.3
C5—C6—H6C	109.5	C19—C18—C17	59.2 (6)
H6A—C6—H6C	109.5	C19—C18—H18A	117.8
H6B—C6—H6C	109.5	C17—C18—H18A	117.8
C4—C7—C8	114.55 (18)	C19—C18—H18B	117.8
C4—C7—H7A	108.6	C17—C18—H18B	117.8
C8—C7—H7A	108.6	H18A—C18—H18B	115.0
C4—C7—H7B	108.6	C18—C19—C17	61.5 (4)
C8—C7—H7B	108.6	C18—C19—H19A	117.6
H7A—C7—H7B	107.6	C17—C19—H19A	117.6
C9—C8—C13	117.5 (2)	C18—C19—H19B	117.6
C9—C8—C7	122.2 (2)	C17—C19—H19B	117.6
C13—C8—C7	120.2 (2)	H19A—C19—H19B	114.7
C8—C9—C10	121.4 (2)	C18'—C17'—C16	113.8 (7)
C8—C9—H9	119.3	C18'—C17'—C19'	61.3 (12)
C10—C9—H9	119.3	C16—C17'—C19'	119.4 (16)
C11—C10—C9	120.9 (2)	C18'—C17'—H17'	116.8
C11—C10—H10	119.5	C16—C17'—H17'	116.8
C9—C10—H10	119.5	C19'—C17'—H17'	116.8
C10—C11—C12	117.7 (2)	C17'—C18'—C19'	63.9 (12)
C10—C11—C14	120.5 (2)	C17'—C18'—H18C	117.4
C12—C11—C14	121.7 (2)	C19'—C18'—H18C	117.4
C13—C12—C11	121.2 (2)	C17'—C18'—H18D	117.4
C13—C12—H12	119.4	C19'—C18'—H18D	117.4
C11—C12—H12	119.4	H18C—C18'—H18D	114.4
C12—C13—C8	121.1 (2)	C18'—C19'—C17'	54.9 (12)
C12—C13—H13	119.5	C18'—C19'—H19C	118.3
C8—C13—H13	119.5	C17'—C19'—H19C	118.3
C11—C14—H14A	109.5	C18'—C19'—H19D	118.3
C11—C14—H14B	109.5	C17'—C19'—H19D	118.3
H14A—C14—H14B	109.5	H19C—C19'—H19D	115.5
C11—C14—H14C	109.5		
			
C2—N1—C1—O1	179.7 (2)	C7—C8—C9—C10	−174.8 (2)
C2—N1—C1—N2	−0.2 (3)	C8—C9—C10—C11	−0.5 (4)
C4—N2—C1—O1	−178.97 (19)	C9—C10—C11—C12	−1.6 (4)
C15—N2—C1—O1	2.1 (3)	C9—C10—C11—C14	179.3 (2)
C4—N2—C1—N1	0.9 (3)	C10—C11—C12—C13	2.3 (4)
C15—N2—C1—N1	−177.95 (17)	C14—C11—C12—C13	−178.6 (2)
C1—N1—C2—O2	178.59 (19)	C11—C12—C13—C8	−0.9 (4)
C1—N1—C2—C3	−1.3 (3)	C9—C8—C13—C12	−1.3 (3)
O2—C2—C3—C4	−177.7 (2)	C7—C8—C13—C12	175.6 (2)
N1—C2—C3—C4	2.2 (3)	C16—O3—C15—N2	−75.0 (2)
O2—C2—C3—C5	2.4 (3)	C1—N2—C15—O3	109.0 (2)
N1—C2—C3—C5	−177.65 (17)	C4—N2—C15—O3	−69.9 (2)
C2—C3—C4—N2	−1.7 (3)	C15—O3—C16—C17	158.4 (2)
C5—C3—C4—N2	178.21 (19)	C15—O3—C16—C17'	−158.1 (4)
C2—C3—C4—C7	179.5 (2)	O3—C16—C17—C19	151.7 (6)
C5—C3—C4—C7	−0.7 (3)	C17'—C16—C17—C19	45.9 (8)
C1—N2—C4—C3	0.0 (3)	O3—C16—C17—C18	83.6 (3)
C15—N2—C4—C3	178.87 (18)	C17'—C16—C17—C18	−22.2 (5)
C1—N2—C4—C7	178.97 (19)	C16—C17—C18—C19	104.5 (6)
C15—N2—C4—C7	−2.2 (3)	C16—C17—C19—C18	−110.9 (4)
C4—C3—C5—C6	−104.2 (2)	O3—C16—C17'—C18'	−79.3 (7)
C2—C3—C5—C6	75.6 (3)	C17—C16—C17'—C18'	10.5 (5)
C3—C4—C7—C8	98.1 (2)	O3—C16—C17'—C19'	−148.5 (14)
N2—C4—C7—C8	−80.8 (2)	C17—C16—C17'—C19'	−58.7 (15)
C4—C7—C8—C9	−28.4 (3)	C16—C17'—C18'—C19'	−111.8 (18)
C4—C7—C8—C13	154.9 (2)	C16—C17'—C19'—C18'	102.6 (12)
C13—C8—C9—C10	1.9 (4)		

### Hydrogen-bond geometry (Å, °)

**Table:**

*D*—H···*A*	*D*—H	H···*A*	*D*···*A*	*D*—H···*A*
N1—H1···O1^i^	0.89 (1)	1.93 (1)	2.816 (2)	178 (2)

Symmetry codes: (i) −*x*+1, −*y*+1, −*z*+1.
